# The Effects of High-Pressure Processing Pre-Treatment on Apple Fruit for Juice Production

**DOI:** 10.3390/foods13142182

**Published:** 2024-07-11

**Authors:** Massimiliano Rinaldi, Rohini Dhenge, Margherita Rodolfi, Paola Littardi, Karen Lacey, Antonella Cavazza, Maria Grimaldi, Veronica Lolli, Martina Cirlini, Benedetta Chiancone, Tommaso Ganino

**Affiliations:** 1 Department of Food and Drug, University of Parma, Parco Area delle Scienze 27/A, 43124 Parma, Italy; massimiliano.rinaldi@unipr.it (M.R.); tommaso.ganino@unipr.it (T.G.); 2 Dipartimento di Scienze Chimiche, della Vita e della Sostenibilità Ambientale, Università degli Studi di Parma, Parco Area delle Scienze 17/A, 43124 Parma, Italy; 3 National Research Council, Institute of BioEconomy (IBE), Via Madonna del Piano, 10-50019 Sesto Fiorentino, FI, Italy

**Keywords:** apple juice, thermal–non-thermal process characterisation, texture, microstructure

## Abstract

One of the most difficult issues in the juice industry is to manufacture juices where processing processes minimise the impact on the native characteristics of the fruits. In this study, high-pressure technology was used on whole apple fruits in order to evaluate the effect on the juice production. Two varieties, cv. Limoncella and cv. Pink Lady, were considered. Preliminarily, the fruits were subjected to different pressures, and histological as well as pomological measurements were taken in order to identify the best treatment condition, which was established to be 600 MPa for 3 min. Juice samples were then characterised by measuring the colour, viscosity, total antioxidant capacity (TAC), and total phenolic content (TPC). The storage colour stability of the juices for both varieties showed not significant L* values between the untreated and pre-treated fruits. Juices obtained from pre-treated fruits had a viscosity significantly higher than that obtained from untreated ones. Interestingly, the TPC of high-pressure processing (HPP) pre-treated juice resulted in being significantly higher compared to the untreated ones. The HPP pre-treatment can be considered as a commercial application to modulate some quality standards for apple juice production.

## 1. Introduction

Apples (*Malus domestica* Borkh.) are one of Europe’s (EU) most popular fruits in terms of harvested products. On a global scale, Italy ranks seventh in global apple production, with 2.25 mL tonnes in 2025, and second in Europe [1]. Globally, apples used for fresh consumption account for 70–75%, while about 30% are processed to various value-added products, including juice, wine, jams, and dried products [2]. Apple juice is one of the most popular products among juices all over the world due to its pleasant sensory qualities, high soluble fibre, and content of dietary phenolic compounds, which are primarily responsible for the fruit’s health benefits [3]. One of the most difficult issues in the juice industry is to manufacture juices with a quality like that of freshly squeezed ones, as well as to ensure a consistent original quality to consumers. As a result, proper processing processes that minimise the impact on native characteristics are essential to provide safe apple products with a high sensory, nutritional, and functional quality. However, some pre-treatments are essential on apple fruits to minimise discolouration, enzyme activity, the loss of nutrients, and weight and colour changes. For instance, Putnik et al. [4] used an ultrasound process on Golden Delicious apples to improve the fruit quality during storage. HPP is a non-thermal technology that is normally used for the purpose of stabilising apple juice to ensure its shelf-life [5,6,7], but now limited works have been conducted using HPP treatments on fruit prior to juice production. Some work has been performed using HPP for the preservation of whole fruits, but the results have shown some problems related to the treatment intensity, which can cause more tissue injuries than blanching, as well as cellular injuries, which have led to the escape of intracellular elements such as bioactive compounds and enzymes. On the other hand, extended treatment times (5 min) showed higher antioxidant activity in whole blackberries treated at 600 MPa, probably due to cell breakage and inner cell fluid leaking [8]. Similarly, De Ancos et al. [9] reported that a pre-treatment at 200 MPa on whole peeled “Navel” oranges was able to produce juices with higher concentrations of phytoene (40%) and phytofluene (10 times) in comparison to those which were untreated. Castro et al. [10] applied high-pressure (HP) treatment on whole green and red pepper fruits (*Capsicum annuum* L.), showing a lower reduction in the level of soluble protein and ascorbic acid content, and, in red pepper, a considerably higher content (+15–20%) of ascorbic acid than in the untreated ones. In accordance with some observations about the texture recovery of cherry tomatoes subjected to high-pressure processing, Tangwongchai et al. [11] observed increasing textural degradation and cell rupture in tomatoes when the pressure was increased up to 400 MPa. The authors explained that it was the result of pectin depolymerisation due to the inactivation of the enzymes, such as polygalacturonase. The authors [11] explained that pressure is involved in textural changes in tomatoes, with at least two related processes. In the first process, cell destruction occurred by the higher compressibility of the gaseous phase (air) compared to the liquid and solid components [11]. The second process implied the release of water due to cell damage caused by enzymatic activity, which caused softening and even more cell damage in the tissues [11]. In confirmation of this, Marigheto and collaborators [12] observed that cell wall damage is apparent at pressures of 300 MPa in fresh strawberries, whereas cell wall swelling and dehydration was observed after HPP (600 MPa) on pineapple [13] and after HPP on vacuum-packed peach (500 MPa) [14]. HPP pre-treatments on fruit for juice production could be an innovative tool to optimise juice extraction, as it can produce an increase in the juice yield due cell wall damage. This observation was previously detected in olive oil production by Andreou and collaborators [15]; in this study, the HPP treatment led to an increase in the olive oil yield by 9.3% compared to a control, confirming the potential beneficial effect in juice extraction.

This work aimed to study, for the first time, the application of high-pressure technology on whole apple fruits in order to evaluate the effect of this treatment on the subsequent juice production process in term of colour, viscosity, total antioxidant capacity (TAC), and total phenolic content (TPC). The study considered two varieties of apple: cv. Limoncella (LIM—an ancient cultivar widespread in central Italy) and cv. Pink Lady (PL—a cultivar widely spread throughout the national territory). The apple juice produced was stabilised with thermal pasteurisation (both treated and untreated) and, for nine months, physical analysis (colour and viscosity) of apple juices were carried out. PL is a late-maturing apple cultivar that originated from ‘Lady Williams’ and ‘Golden Delicious’ [16]. This cv. is being planted in various apple-producing areas of the world owing to its great flavour and sensory qualities. The shape of PL apples is oblong–conical, with broad regions of green–yellow and solid pinkish-red skin; the skin is thin, lenticels usually are not conspicuous, and the flesh is white, dense, firm, moderately juicy, sweet with an acid balance, and is a late-maturing, long-storing variety that can be harvested after the conventional late season [16]. LIM apples, commonly known as ‘*Limone*’ (lemon) apples, are a cultivar that is widespread in central–southern Italy [17]. The skin of LIM apples acquires a green–yellow colour, it is thick, rather wax-like, and possesses numerous rust lentils [18]. In addition, LIM apples are characterised by a peculiar citrus flavour. 

## 2. Materials and Methods

### 2.1. Samples and Storage

Freshly harvested apples belonging to two cultivars, Pink Lady^®^ (PL) and Limoncella (LIM) were kindly donated by Azienda Agricola Polidoro Sandro, Ortona (CH), Italy. The apples were harvested in October (LIM) and November (PL), and immediately transported in the laboratory under refrigerated conditions, then stored at 4 °C until use. The apples were processed 2 weeks after storage. After the removal of the stalks, whole fruits were vacuum-packed in plastic bags (OPA/PP 15/65, Orved, Musile di Piave, Italy) with a thickness of 80 μm and an oxygen and water vapour permeability of <35 cm^3^/m^2^/24 h and 3.3 g/m^2^/24 h, respectively.

### 2.2. Choice of the Best Processing Condition and Apple Fruit Characterisation

#### 2.2.1. High-Pressure Processing (HPP)

HPP was performed at the enterprise JBT Italia (Parma, Italy) using 30 L equipment (Avure Technologies Middletown, OH, USA) with a come-up time of 200 MPa·min^−1^. Different treatment intensities were carried out for the determination of the best condition to use in the following experiments. The pressures employed in the HPP were 200, 400, and 600 MPa for 180 s. The processes were conducted using cold water (4–5 °C) as the pressure medium to keep the temperature of the system around 18–20 °C, despite the temperature increasing due to pressurisation. Some treated samples were used for the physical analysis and preserved in FAA solutions (FAA = formalin: acetic acid: 60% ethylic alcohol solution, 2:1:17 *v*/*v*) for the microstructure analysis. The rest of the treated fruits were used for juice production. A total of 10 bags containing 6 fruits each were used for each treatment.

#### 2.2.2. Histological Analysis of Apple Fruits

LIM and PL samples, Untr (untreated) and treated at 200, 400, and 600 MPa for 180 s, were cut into small cubes (15 mm side) and placed in FAA solution [19] to block the structural changes. Subsequently, the samples were dehydrated by immersing them in solutions with increasing concentrations of alcohols. The inclusion was made in a methacrylate resin (Technovit 7100, Heraeus Kulzer & Co., Wehrheim, Germany). The blocks obtained were cut using a microtome (Leitz, Wetzlar, Germany), obtaining transversal sections with a thickness of 3 µm. The sections were stained with a solution of toluidine blue (TBO) to observe the general structure. The observations were made using a Leica DM 4000 optical microscope (Leica Imaging Systems Ltd., Wetzlar, Germany) equipped with a Leica DC 100 digital camera. Six replicates were made for each sample.

#### 2.2.3. Physical Analysis of Apple Fruits

Pomological properties (fruit weight, volume, transverse diameter, and longitudinal diameter) and juice yield were determined on both the Untr and HPP apple samples. The measurements were carried out on 10 fruits per each variety, while the juice yield was determined using about 1 kg of fruit for each measurement. The measurements were performed in triplicate. 

#### 2.2.4. Texture Evaluation of Apple Fruits

The texture of the LIM and PL samples, Untr and treated at 200, 400, and 600 MPa for 180 s, was analysed using a TA.XT2 Texture Analyzer equipped with a 245.2 N load cell (Stable Micro Systems, Godalming, UK), a force resolution equal to 0.01 N, and an accuracy value of 0.025%. A puncture test was performed on the equatorial part of the fruit using a 3 mm diameter cylinder probe at a speed of 1 mms^−1^, obtaining the maximum penetration force (hardness, N) from force vs. time curves. For all of the tests, 10 fruits were analysed for each treatment.

#### 2.2.5. Colour, Sucrose, Malic Acid, and Epicatechin Contents of Apple Measurements

The colorimetric measurements of apple samples, subjected to different pressure intensities, were performed with a Minolta CM-2600d colorimeter (Konica Minolta Co., Osaka, Japan) equipped with Spectramagic 3.6 software, a D65 illuminant, and with a measuring angle of 10° compared to normal. The degree of lightness is represented by the L* value, which ranges from 0 (black) to 100 (white). The a* value represents the degree of greenness (negative) to redness (positive), whereas the b* value represents the degree of blueness (negative) to yellowness (positive), respectively. The colour identification was performed on both the peel (at different locations) and the pulp surface of apples from each sample at room temperature. A total of 10 measurements were taken for each sample.

The sucrose, malic acid, and epicatechin contents of the apple samples were measured by means of the ^1^H NMR spectroscopy method, as reported by Dhenge et al. [20].

### 2.3. Target Quality Analyses of Apple Juice

#### 2.3.1. Juice Extraction and Thermal Pasteurisation (TP)

**Figure 1:**
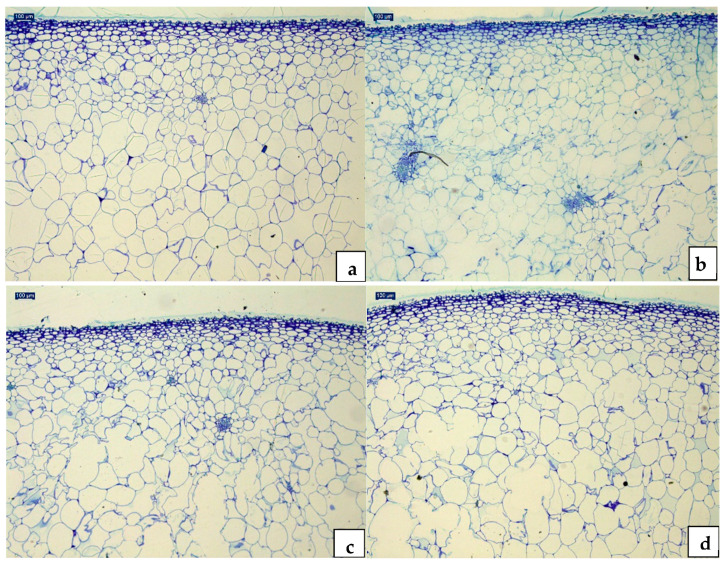
Transverse sections of apple samples subjected to HPP treatments at different pressures and stained with toluidine blue: (**a**) Untreated (20×); (**b**) HPP200 (20×); (**c**) HPP400 (20×); (**d**) HPP600 (20×). Damages (fissures) are visible in (**b**–**d**). Increasing the intensity of the HPP treatments increase the tissue damage (see figure (**d**)).

Juices were obtained from untreated (Untr) and high-pressure-treated (HPP) fruits. Only the fruits treated at 600 MPa were used for the juice extraction; this choice was based on histological results (Figure 1), as, at this condition, the fruits showed the highest changes. Apple juice extraction was performed using a domestic slow juicer (Hotpoint, Peterborough, Cambridgeshire, UK) with a rotation speed of 70 rpm and a sieve dimension of 500 µm. To avoid juice oxidation, as in the current industrial practice, 0.5 mg/kg of ascorbic acid was added to the juice just after the extraction [6]. Fresh juices (from the Untr and HPP apples) were filled in glass jars with metallic cap and pasteurised by immersing them in water at 90 °C for 4 min [21] and stored up to 9 months at room temperature.

#### 2.3.2. Colour Measurement of Juices

The colorimetric measurement of the apple juice samples was performed as previously reported in Section 2.2.5. Subsequently, the apple juice samples were placed in a cell and covered with a white plate, and each sample was analysed with a total of 10 replicates.

#### 2.3.3. Viscosity of Juices

The rheological parameters of the juices were determined in triplicate for each sample with the rotational rheometer ARES-TA^®^ (Advanced Rheometric Expansion System, TA Instruments, New Castle, DE, USA) coupled with Orchestrator TM software v7.2.0.4. The study was performed at 25 °C and at shear rates between 10 and 300 s^−1^ with the Couette geometry (concentric cylinders) with the following dimensions: diameter of the cup = 34 mm, concentric cylinder diameter = 32 mm, and length = 33 mm. 

#### 2.3.4. Total Antioxidant Capacity (TAC) and Total Phenolic Content (TPC)

The analysis of the total antioxidant capacity was carried out using the DPPH (2,2-diphenyl-1-picrylhydrazyl) method [22,23]. For the analysis, 1 mL of apple juice was centrifuged at 12,000× *g* for 15 min at 4 °C. The supernatant was collected and used for the DPPH assay. The solution of DPPH (2,2-diphenyl-1-picrylhydrazyl) was prepared at a concentration of 0.2 mmol·L^−1^ in 70% methanol. The blank for the spectrophotometric analysis was prepared as follows: 0.2 mL of distilled water + 4.6 mL of 70% methanol. The samples were prepared as follows: 0.2 mL of extract + 2 mL of DPPH solution + 2.6 mL of 70% methanol. The % quenching of DPPH was calculated using the following equation:

*I* (%) = (*A_DPPH_* − *A_S_*) × 100/*A_DPPH_*

where *I* is the percentage of inhibition, *A_DPPH_* is the absorbance of the blank, and *A_S_* is the absorbance of the solution containing the apple juice, measured at a wavelength of 517 nm.

For the TPC analysis, an aliquot (50 μL) of the juice extract was added to 1160 μL of water (MilliQ), 300 μL of sodium carbonate 20% *w*/*w* to ensure the optimum pH for the formation of phenolate ions [24], and 100 μL of the Folin–Ciocalteu reagent; the solution was then incubated at 40 °C for 30 min. An identical preparation of the blank was performed but lacked the sample. Absorbance was measured at 760 nm. The TPC value was expressed as mg of GAE (gallic acid equivalent)/g of dry sample. The calibration curve was generated using 7.5 to 125 µg·mL^−1^ gallic acid. All of the analyses were repeated in triplicate.

### 2.4. Statistical Analysis

For all data, means and standard deviations were calculated with SPSS (v. 27.0, SPSS Inc., Chicago, IL, USA). Significant differences between the data were verified by Student’s *t-*test and by one-way analysis of variance (ANOVA) followed by Tukey’s post hoc test at *p* < 0.05 by using the same software. 

## 3. Results

### 3.1. Choice of the Best Processing Condition and Apple Fruit Characterisation

#### 3.1.1. Microstructure Observation and Treatment Evaluation

The microstructural examination of apple tissues by the optical microscope revealed the HPP treatment intensity that produced the higher damage at the tissue level and, thus, that could enhance the fruit juice extraction. In the microscope observations, distinct patterns of parenchyma cells were observed. The structural components are distinguishable from each other, except for the single layer of epidermal cells located directly under the cuticle and vascular bundles surrounded by small parenchymatic cells (Figure 1). 

The untreated samples showed an epidermis with cells ranging in size from 15 to 20 µm, below which two smaller cell layers were observed. The observation of the innermost layers has shown how the cell dimensions gradually become larger up to varying sizes between 150 and 250 µm. The larger size of the cells and the rounded shape determine the formation of large intercellular spaces as well as regular arrangements (Figure 1a). The histological results of the two apple varieties are similar, and for this reason, only the images of the apple cv. LIM are shown. The microscopic analysis of the LIM and PL apples treated at 600 MPa for 180 s showed how the epidermis remained intact; the analysis of the deeper layers, on the other hand, showed a progressive increase in cell detachment, and, in the deeper layers, the rupture of the cell wall, with a consequent increase in intercellular spaces and deep gap formations (Figure 1b). In the reserve parenchyma, which contains starch granules (as observed under the microscope using Lugol’s reagent), several gaps were found, caused by the rupture of the cell walls, and, in addition, in some intercellular spaces, there are inclusions of unknown origin, presumably due to the overflow of the cell contents after the rupture (Figure 1c). These structural changes could be due to the more intense gelatinisation of the starch and pectin present in the apple, induced by the treatment at 600 MPa. Briones-Labarca et al. [25] conducted studies on peeled apples treated at 500 MPa for 4, 8, and 10 min. The results revealed that resistant starch increased by 27, 76, and 84%, whereas the digestible starch significantly increased consequently. Similarly, in a study on the effect of HPP treatments in pumpkins, Paciulli et al. [26] observed that the quantity of starch granules and starch gelatinisation depends on the intensity of pressure and time.

In order to improve the physical and chemical quality of apple juices, the HPP condition that damaged the most fruit tissues were chosen. The choice (HPP at 600 MPa) is supported by the concept that greater tissue damage corresponds to a greater extraction capacity [9,27], and therefore it is expected to have better juice extracts with valuable nutritional and physico-chemical properties.

#### 3.1.2. Physical Analysis of Apple Fruits

Both for the LIM and PL apples, the HPP treatments produced changes in the pomological traits, with a significant reduction in the weight, volume, transversal, and longitudinal diameter compared to the Untr ones (Table 1), and a loss of liquid in the bags was observed. This loss of cellular fluid also caused a significant decrease in the juice yield (%) of both cultivars after high hydrostatic pressure treatments (Table 1), as the liquid inside of the bags was discarded before the weight measurements and was not considered as juice.

#### 3.1.3. Texture Profile of Apple Fruits

Regarding the texture of the fruits, for both varieties, the Untr apples appeared to have a statistically harder texture than the HPP-treated ones (Table 1), and this texture difference was caused by the microstructural modifications described in Section 3.1.1.

The correlation between the reduction in the hardness and HPP treatment was also previously observed by Tangwongchai et al. [11], Denoya et al. [14], Rinaldi et al. [13], and Paciulli et al. [26] in other fruits. On the contrary, Kaushik et al. [28] reported an increase in firmness for litchi fruits after high-pressure treatments, but, in that case, the samples were treated at lower pressures.

#### 3.1.4. Colour, Sucrose, Malic Acid, and Epicatechin Contents of Apple Fruits

**Figure 2:**
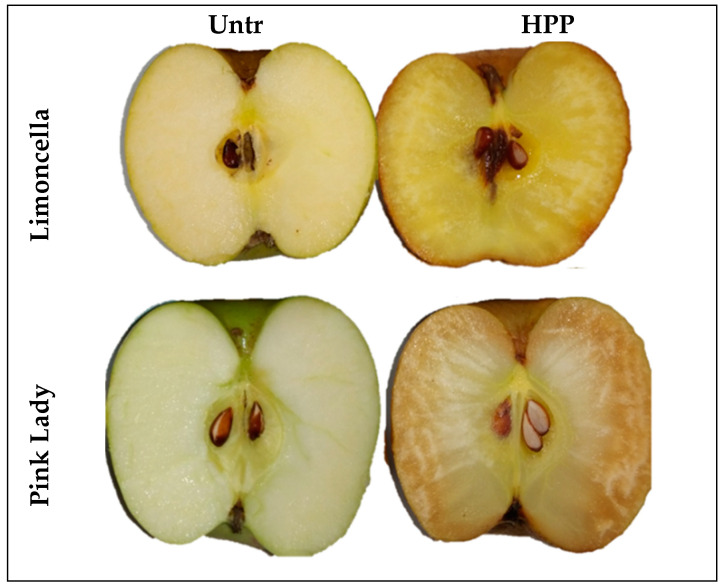
Pictures of apples cv. Limoncella and cv. Pink Lady before (Untr) and after treatment (HPP).

The colorimetric data of the apples are reported in Table 2. In general, PL presented brighter and greener colours compared to LIM, as depicted in Figure 2. Confirming the visual appearance, the pulp of all HPP samples was darker than the Untr ones. The HPP apples showed a highly significant reduction in L* and an increase in a* towards a darker colour. On the contrary, b* did not present significant variation for PL, while a significant decrease in yellow colour was observed in LIM (Table 2). The browning of the apple products after the HPP treatments was reported due to higher PPO reaction rates, since substrates are more available for the enzymes after HPP and/or reactivation of browning enzymes may occur [29]. Colour variations in the peel were significant between the treated and untreated samples only for the LIM samples, with the same trend observed for the pulp. In the PL samples, the peel colour was not uniform and high variability was observed.

The contents of sucrose and malic acids were higher in the PL Untr and PL HPP samples than in the LIM. Instead, epicatechin, which is one of the most important phenols in apple fruits [30], showed a higher content in LIM than PL (Table 2).

The results indicate that both the sucrose and malic acid contents of the apples were not affected by HPP (Table 2), as per Barba et al. [31], which reported that total soluble solids and titratable acidity were not significantly affected by HPP. On the contrary, epicatechin showed a significant increase after the HPP treatment in PL and LIM. In general, epicatechin is reported to be mainly located in the peel and seeds [32], and probably the HPP process could have increased its release from them due to a mechanical effect or physical damage.

### 3.2. Target Quality Analyses of Apple Juice

#### 3.2.1. Colour Measurement

Despite the significant differences in the pulp and peel colour (Table 2), the juices obtained, starting from the HPP fruits, did not present significantly differences in L* values (Table 3), neither at day 0 nor after 9 months of storage, for both varieties. In general, the evolution of the colour during 9 months of storage did not seem different in the juices obtained from the HPP apples compared to the untreated ones; probably, the compounds extracted during juicing were more or less the same and, thanks to the thermal treatment, the enzymes were inactivated to a sufficient extent. Nonetheless, it was observed that the colour parameters a* increased during storage, while b* decreased with time.

Significant differences were observed for a* and b*, with lower values for both colorimetric parameters in the juices obtained from the HPP apples compared to the Untr apples. Thus, the juices from the HPP fruits were greener (more negative a* value) and less yellow (lower b* values), and the thermal treatment used for the stabilisation did not cause modifications that were able to cover differences between the juices. The results disagree with Xu et al. [33], who used HPP as a pre-treatment on persimmon and reported no significant changes in juice colour after a pre-treatment at 300 MPa, demonstrating that different fruit compositions and different pressures could give different results.

#### 3.2.2. Viscosity

**Figure 3:**
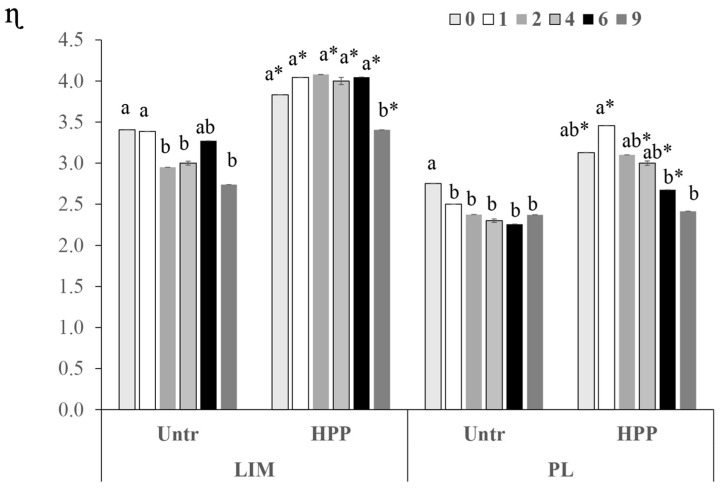
Viscosity (ɳ) expressed in mPas at 100 s^−1^ of cv. Limoncella (LIM) and cv. Pink Lady^®^ (PL) apple juice at 25 °C during 9 months of storage at 4 °C. Values are shown as mean ± standard deviation. Different letters, within times for each sample, indicate statistically different values (*p* < 0.05). * significant differences between treated (HPP) and untreated (Untr) samples at the same time (* *p* < 0.05).

From the statistical analysis, it is evident that the viscosity of the juices obtained from pre-treated fruits was significantly higher than that of the untreated apples during storage time (Figure 3). The resultant viscosity of the juices with pre-treated fruits increased at time 0 day by about 10% due to the disruption of the cell wall, favouring the increase in the starch gelatinisation and pectin solubilisation [27]. The residual activity of enzymes such as polygalactouranase (PG) is another reason for this trend in the juice viscosity.

The pressure-induced release of pectin methylesterase results in the demethylation of pectin, further leading to the formation of low methoxy pectin forming gel networks with the divalent ions, such as Ca^2+^ present in fruits and vegetables [34]. Another reason cited for the increase in the viscosity is the possibility of protein–tissue coagulation to thicken the juice texture [35]. This hypothesis is confirmed by the results of Xu et al. [33], who reported that the soluble pectin content in the persimmon juice obtained from HPP pre-treated fruits decreased significantly due to the enzymatic hydrolysis of the pectin or the formation of a pectin–tannin complex.

The viscosity of the LIM juice was higher compared to PL due to the higher solid content, and the LIM juice after the HPP pre-treatment seemed to be more stable over time than PL (Figure 3). The HPP pre-treatment could be a potential way to improve the viscosity and viscosity stability during storage.

#### 3.2.3. Total Antioxidant Capacity (TAC) and Total Phenolic Content (TPC)

**Figure 4:**
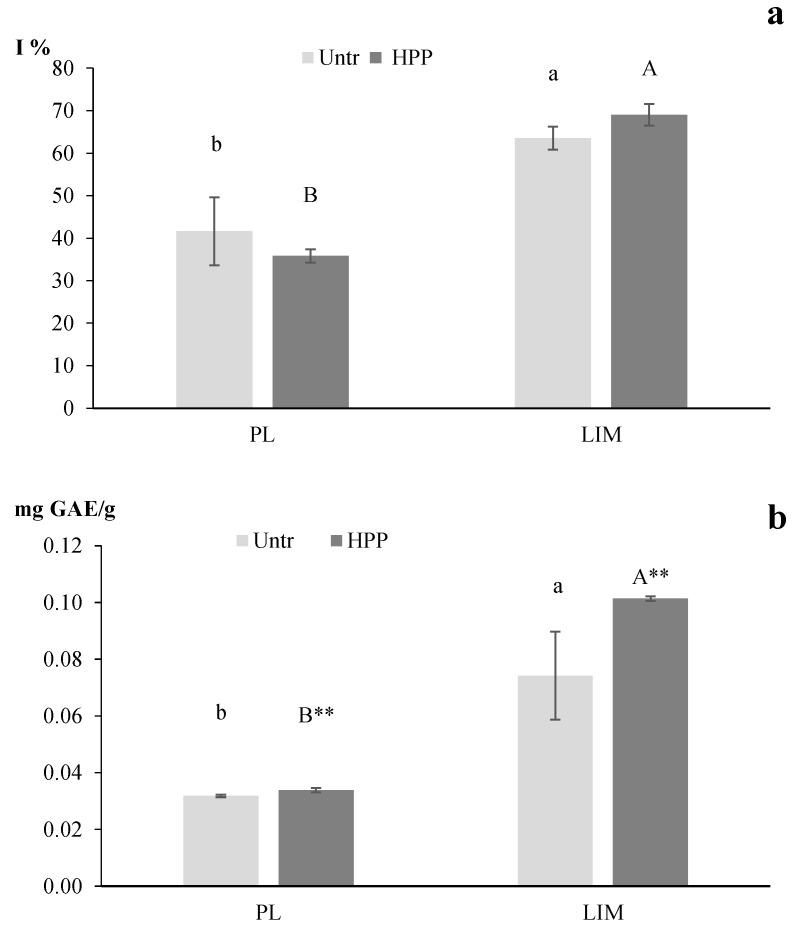
Total antioxidant capacity (TAC) (inhibition %, I%) (**a**) and total phenolic content (TPC) (mg GAE\g) (**b**) of apple juice obtained using apple fruits of the cv. Limoncella (LIM) and cv. Pink Lady^®^ (PL) subjected (or not) to HPP treatment. Different lowercase letters indicate statistically different values (*p* < 0.05) between different Untr varieties. Different capital letters indicate statistically different values (*p* < 0.05) between different HPP varieties. ** significant differences between treated (HPP) and untreated (Untr) samples of the same cultivar (** *p* < 0.01).

The resulting TAC as well as the TPC of the LIM juices was higher than the PL ones (Figure 4), which was in accordance with the data previously shown concerning the epicatechin content (Table 2) and with the results reported by Francini and Sebastiani [32]. In this study, the authors reported LIM apples characterised by a higher phenolic concentration when compared to other commercial apple cultivars. For both varieties, the TAC values resulted in being not significantly different between the HPP and Untr juices, probably because of the limited differences in the compounds that react with DPPH. Interestingly, the TPC of both the LIM and PL HPP juice resulted in being significantly higher compared to Untr one (Figure 4) following the epicatechin increase (Table 2). These results are in accord with the study of De Ancos et al. [9], where an increase in flavonoids and vitamin C content for Navel oranges pre-treated by HPP before juicing were observed. No clear correlation or trend between the TAC and TPC was observed. Similarly, Imeh and Khokhar [36] reported a weak correlation between the TPC and TAC in fruits, comprising apples. Finally, the obtained results on apples could be encouraging in studying HPP pre-treatment as an effective treatment to valorise ancient cultivars and obtain juices with higher TPC than the corresponding freshly prepared juices.

## 4. Conclusions

The effects of different intensities of the HPP pre-treatment on whole apples intended for juice production were evaluated, taking into consideration the apple microstructure. The treatment at 600 MPa was chosen for the subsequent analysis on the pomological properties, colour, texture profile in the whole apple and viscosity, antioxidants, and colour of the LIM and PL apple juices thereof. From the results obtained in the present study, it can be concluded that HPP resulted in a significant change in the measured pomological characteristics (weight, volume, height, and width), juice yield (%), and hardness (N). The Untr apples exhibited a better retention of the natural apple juice colour compared to the HPP apples, while the sucrose in both cultivars and malic acid content in PL were not affected by the HPP treatment. On the contrary, a significant increase in epicatechin for both varieties was observed after the HPP treatment of the whole fruit, which is one of the most important phenols in apples. This increase could be due to the microstructural changes and release of this compound due to mechanical and physical damages. The HPP juice showed a significantly higher viscosity than the Untr juice, probably due to starch gelatinisation and pectin solubilisation from the cell wall breakdown. The TAC values resulted in being not significantly different but, interestingly, the TPC of the HPP LIM and PL juices resulted in being significantly higher compared to the untreated one. The experimental results proved that HPP could be an effective pre-treatment on apples and can be considered as a commercial application to modulate some quality standards for apple juice production.

## Figures and Tables

**Table 1:** Influence of HPP treatments on pomological and textural properties of cv. Limoncella (LIM) and cv. Pink Lady^®^ (PL) apples.

Samples	Weight (g)	Volume (ml)	Transversal Diameter (cm)	Longitudinal Diameter (cm)	Juice Yield (%)	Hardness (N)
	**LIM**					
Untr	166.5 ± 13.4 a	205.6 ± 13.3 a	7.21 ± 0.14 a	6.86 ± 0.35 a	70.8 ± 5.8 a	21.4 ± 2.3 a
200 MPa	157.3 ± 14.7 b	160.0 ± 0.3 b	6.36 ± 0.32 b	6.37 ± 0.32 b	54.3 ± 3.4 b	18.1 ± 1.3 b
400 MPa	156.9 ± 9.4 c	162.7 ± 10.9 b	6.48 ± 0.36 b	6.15 ± 0.36 b	54.9 ± 3.5 b	17.9 ± 1.9 bc
600 MPa	156.1 ± 15.1 c	155.7 ± 13.06 c	6.33 ± 0.25 b	6.28 ± 0.43 b	49.5 ± 3.3 c	16.8 ± 1.2 c
	**PL**					
Untr	159.5 ± 11.3 a	198.5 ± 14.1 a	6.18 ± 0.16 a	7.82 ± 0.39 a	67.8 ± 4.7 a	19.5 ± 1.7 a
200 MPa	146.3 ± 09.2 c	154.9 ± 0.2 bc	5.33 ± 0.25 b	5.34 ± 0.41 b	57.2 ± 2.3 b	15.2 ± 1.6 b
400 MPa	148.8 ± 9.1 b	159.8 ± 08.7 b	5.42 ± 0.43 b	4.16 ± 0.33 b	49.8 ± 2.5 c	13.9 ± 0.85 bc
600 MPa	144.1 ± 13.0 bc	151.8 ± 11.8 c	5.31 ± 0.19 b	5.27 ± 0.47 b	51.5 ± 2.3 bc	11.5 ± 1.23 c

Values are shown as mean ± standard deviation. Different letters, within each column, indicate statistically different values (*p* < 0.05).

**Table 2:** Influence of high pressures on the colorimetric and chemical parameters of cv. Limoncella (LIM) and cv. Pink Lady^®^ (PL) pulp and peel.

	LIM	PL		
	Untr	HPP	Untr	HPP
	**Pulp**			
L*	78.8 ± 2.3	50.0 ± 2.2	83.6 ± 1.1 **	56.4 ± 2.2
a*	3.9 ± 1.1	6.0 ± 1.3	−1.4 ± 0.4 **	7.7 ± 1.0
b*	30.0 ± 4.6	23.0 ± 1.4	22.7 ± 2.1	19.7 ± 2.1
	**Peel**			
L*	73.2 ± 2.0 **	53.0 ± 2.8	65.3 ± 5.2	51.6 ± 6.4
a*	0.2 ± 3.3 *	7.6 ± 2.3	4.1 ± 6.7	8.9 ± 6.9
b*	42.7 ± 4.3 **	20.7 ± 3.4	25.5 ± 5.7	19.4 ± 4.9
Sucrose (g/100mL)	7.7 ± 1.2	7.9 ± 0.9	13.4 ± 0.9	13.1 ± 1.3
Malic acid (g/100mL)	0.65 ± 0.06 *	0.59 ± 0.06	2.32 ± 0.22	2.14 ± 0.11
Epicatechin (mg/100mL)	51.6 ± 3.9	54.5 ± 2.7 *	11.2 ± 1.9	20.8 ± 1.5 *

*,** significant differences between treated (HPP) and untreated (Untr) samples (* *p* < 0.05; ** *p* < 0.01).

**Table 3:** Colorimetric parameters of cv. Limoncella (LIM) and cv. Pink Lady^®^ (PL) apple juices obtained from untreated (Untr) and treated (HPP) fruits during 9-month storage at 4 °C.

	*LIM*					
	Untr			HPP		
Time (months)	L*	a*	b*	L*	a*	b*
0	44.6 ± 2.3 a	−1.58 ± 0.03 c	16.6 ± 0.1 a	43.3 ± 0.2 a	−1.71 ± 0.01 ab*	14.3 ± 0.52 a*
1	44.5 ± 1.8 a	−1.56 ± 0.09 c	15.7 ± 0.9 b	43.6 ± 0.8 a	−1.78 ± 0.03 ab*	14.3 ± 0.4 a*
2	44.4 ± 1.3 a	−1.42 ± 0.05 c	14.9 ± 0.6 bc	43.6 ± 1.1 a	−1.63 ± 0.02 a*	14.2 ± 0.1 a
4	44.2 ± 1.0 a	−1.20 ± 0.05 b	13.3 ± 1.0 c	44.0 ± 0.8 a	−1.65 ± 0.06 a*	13.9 ± 0.7 ab
6	44.0 ± 2.2 a	−0.71 ± 0.02 a	13.6 ± 0.3 c	44.2 ± 0.3 a	−1.86 ± 0.12 b*	13.3 ± 1.1 ab
9	43.7 ± 1.1 b	−1.21 ± 0.04 b	14.3 ± 0.3 bc	42.5 ± 1.0 b	−1.62 ± 0.06 a*	12.0 ± 1.1 b*
	***PL***					
	**Untr**			**HPP**		
	L*	a*	b*	L*	a*	b*
0	42.1 ± 0.3 a	−2.21 ± 0.03 c	7.1 ± 0.1 b	40.0 ± 1.1 ab	−2.14 ± 0.02 bc	5.1 ± 0.21 c*
1	42.0 ± 0.8 a	−2.00 ± 0.02 c	7.1 ± 1.1 b	41.6 ± 0.8 ab	−2.34 ± 0.06 c*	5.2 ± 0.8 c*
2	41.9 ± 0.5 a	−1.79 ± 0.04 b	7.1 ± 1.3 b	40.6 ± 1.9 ab	−2.43 ± 0.01 c*	5.3 ± 0.9 c*
4	41.8 ± 1.3 a	−1.41 ± 0.01 b	7.0 ± 1.0 b	39.6 ± 1.8 b	−2.37 ± 0.05 c*	5.6 ± 0.5 b*
6	41.6 ± 0.2 a	−1.07 ± 0.03 ab	6.9 ± 1.2 b	43.2 ± 0.7 a	−1.92 ± 0.02 b*	5.7 ± 1.3 b*
9	41.4 ± 1.8 a	−0.61 ± 0.06 a	7.7 ± 1.3 a	38.4 ± 0.8 b*	−0.56 ± 0.08 a	6.2 ± 1.0 a*

Values are shown as mean ± standard deviation. Different letters, within each column, indicate statistically different values (*p* < 0.05). * significant differences between treated (HPP) and untreated (Untr) samples at the same time (* *p* < 0.05).

## Data Availability

The original contributions presented in the study are included in the article, further inquiries can be directed to the corresponding author.
